# Intersecting inequities: a scoping review of the gendered relationship between unpaid care work and intimate partner violence during the COVID-19 lockdown in Canada

**DOI:** 10.1080/16549716.2024.2411743

**Published:** 2024-10-18

**Authors:** Alexandra Beukens, Julia Smith

**Affiliations:** Faculty of Health Sciences, Simon Fraser University, Burnaby, BC, Canada

**Keywords:** Pandemic, women, caregiving, intimate partner violence, policy

## Abstract

**Background:**

While there is now extensive research on how COVID-19 lockdowns negatively affected unpaid care burdens and intimate partner violence (IPV), the structural determinants shaping both experiences are less well understood.

**Objectives:**

The review seeks to answer: how did structural determinants of gender inequality shape both the experiences of increased unpaid care burdens and IPV during the COVID-19 pandemic lockdown? Which policy proposals might mitigate these effects during future pandemic preparedness and response?

**Methods:**

We conducted a scoping review of two sets of literature: on COVID-19 and unpaid care and COVID-19 on IPV. Following systematic searches of key databases and the application of inclusion/exclusion criteria, we analyzed articles using a gender matrix framework to identify common themes and policy recommendations.

**Results:**

Common themes include adherence to traditional gender norms, power dynamics featuring coercive control, narrowed pathways to formal and informal supports, and compounding emotional tolls. Policy recommendations from the literature aimed at addressing structural determinants of gender inequality common to both unpaid care and IPV, including expanded access to virtual support services, workplace policies that value the contributions of caregivers, enhanced engagement efforts to incorporate intersectional understandings, and funding for caregiver support services and the anti-violence sector which recognize the value of their contributions.

**Conclusions:**

Enhanced understanding of the structural determinants of gender inequality at play in experiences of unpaid care work and IPV highlights gaps in pandemic response, which overlooked the role of gender inequities in shaping relationship dynamics, as well as areas for more gender transformative policies.

## Paper Context

- **Main findings:** Literature on the experiences of unpaid care and intimate partner violence during COVID-19 underscore common experiences of structural determinants of gender inequality.
- **Added knowledge:** This research draws attention to shared gendered dynamics at the heart of the two experiences, including adherence to traditional gender norms, power dynamics featuring coercive control, narrowed pathways to resources, and compounding emotional tolls.
- **Global Health Impact for Policy and Action:** Specific policy interventions, paired with social change movements, can target the structural determinants of gender inequality which shape both experiences.

## Background

As soon as the COVID-19 pandemic was declared in March 2020, early predictions suggested that public health measures put in place to mitigate the spread of the virus, such as stay-at-home orders and the closure of various social services, would lead to specific gendered impacts, including an increase in intimate partner violence (IPV) and a disproportionate shift of unpaid care responsibilities to women [1,2]. Similar gendered impacts have been observed in past disease outbreaks, such as the Ebola epidemic in West Africa and the Zika virus in South and Central America [3,4]. With the official Public Health Emergency of International Concern now over, ample evidence demonstrates these predictions were, unfortunately, accurate around the world. COVID-19 saw a deepening of gender-based labour divisions and an increase in the burden of unpaid care work falling to women [5,6]. While households around the world adjusted to the new realities of living through a pandemic and spent more time at home, rates of IPV also increased [7].

Canada provides a critical case to explore the gendered effects of the pandemic, as early global assessments of Canada’s pandemic response have celebrated it for centering gender-based analysis [8]. The Canadian federal government described its COVID-19 response as ‘feminist’ in budget and related documentation, including specific measures and funding to address IPV and unpaid care [9]. However, the national COVID-19 response must be situated in a context of persistent gender inequalities. The dominant colonial settler culture in Canada remains informed by patriarchal traditions, which in turn influence social and legal historical precedents [10]. This reality positions men as public-facing leaders, while women remain associated with the private sphere, despite almost equal labour participation [10]. For example, a recent Statistics Canada report showed that women spend, on average, 3.9 h per day on unpaid domestic labour compared to 2.4 h for men [11]. This same survey showed that 60.8% of women did unpaid care work in addition to a paid role, while the same was true for only 40.2% of men [11]. Though IPV can be perpetrated against people of any gender, statistics indicate that women are disproportionately affected. In Canada, four out of five cases of police-reported IPV are perpetrated against women [12]. Hesitancy to report cases of IPV partially speaks to the societal normalization of gendered power discrepancies within relationships and gender-based barriers to help-seeking behaviour [13]. Responses to a 2014 Canadian social survey indicate that only 19% of spousal violence victims file a police report [14]. This tension – between stated policy intentions and persistent inequities – provides a critical context through which to explore the underlying drivers of the gendered effects of the pandemic.

While experiences of unpaid care and IPV during the COVID-19 pandemic have been well documented, the structural determinants of gender inequality connecting them have not. By structural determinants of gender inequality, we refer to the socioeconomic, cultural and political processes that structure hierarchical power relations and shape the environments that facilitate or impede people’s livelihoods and security [15]. More often, the gendered effects of pandemics are analyzed in isolation, limiting the understanding of their determinants, as well as of comprehensive strategies to address the multiple gendered effects of pandemic response. In order to advance understanding of the relationship between increased unpaid care burdens and IPV experienced by women, we conducted a scoping review of the literature aimed at exploring the gendered determinants that shaped women’s experiences during the initial months of the COVID-19 pandemic in Canada, when public health guidelines were most restrictive and therefore had acute social effects. This review’s objective is to illuminate the structural determinants of gender inequality common across the experiences of increased unpaid care burdens and IPV during the COVID-19 pandemic lockdown and identify policy proposals that may mitigate these effects during future pandemic preparedness and response. We explore four key overlapping themes identified in the literature and conclude with policy recommendations from the literature.

## Methods

We conducted two searches in February 2023 (one on unpaid care during the COVID-19 lockdown and the other on IPV during the COVID-19 lockdown) in an iterative scoping review methodology as described by Arksey & O’Malley [16]. This approach involves the stepwise development of a research question, identification of relevant databases, development of a search strategy, study selection, full-text review, data extraction using a descriptive analytical method, and theme analysis [16]. Notably, it involves the wide-reaching incorporation of data, including that from both quantitative and qualitative research [16]. As the aim was to develop a broad but complex understanding of two different fields of study (i.e. IPV and unpaid care) and to identify gaps in pandemic response, such a scoping review was determined to be the most appropriate method to achieve this.

The search strategy was arrived at by the authors following familiarization with published literature and terminology used in the Canadian context to refer to experiences of caregiving and IPV. Subject headings were used to develop key search terms. For example, the subject heading of IPV included ‘IPV’, ‘domestic violence’, ‘partner abuse’ ‘intimate partner aggression’, ‘gender-based violence’, ‘gbv’ … (see Appendix A for list of all terms). Databases searched included Google Scholar, PubMed, CINAHL Complete, and Web of Science with search terms applied to All Fields in each database. As we wished to focus on experiences of caregiving occurring in home environments, we employed the Boolean NOT operator for this specific search, ‘NOT “healthcare”, “health services”, “hospital”, “health facilities”, “clinical”’ to filter out research situated in healthcare settings. To manage the plethora of the literature that has been published on the topics of unpaid care and IPV during COVID-19, we have limited our search to the Canadian context and articles published in English. We only included articles published in peer-reviewed journals as a means to ensure minimum quality. In addition, we limited our search to articles documenting effects from March to June 2020, as this was the time period in Canada when most businesses, schools and services were closed, and physical distancing measures were in place. Articles which examined experiences of unpaid care and IPV beyond the COVID-19 lockdown from March to June 2020 were requested for full-text review and included in cases where the authors detected a strong focus on experiences in the midst of strict stay-at-home orders. Inclusion criteria further included a specific focus on women’s experiences of either unpaid care or IPV. While we acknowledge that people of other genders also experienced both effects – and that sexual and gender minorities are also particularly put at risk of IPV [17,18] – here we focus on women, as the evidence suggests a disproportionate burden of both effects on this gender [11,12]. Exclusion criteria included those articles examining forms of violence other than that between two intimate partners or those who had formerly been intimate partners and those not regarding the provision of unpaid care within private dwellings.

Title and abstract screening was conducted in Covidence by one author, with any uncertainties reviewed by the second author. Article screening was completed according to the PRISMA Extension for Scoping Review guidelines [19]. Articles which appeared relevant to the research questions from their title and abstract and which aligned with the inclusion criteria detailed here in an Appendix A were set aside for full-text review. In cases where an article’s relevance to the research questions was ambiguous, full-text articles were also requested for further screening.

Information extracted from articles following the full-text review included geographic location, type of study, main conclusions, study population, policy suggestions, and any implicit or explicit mention of IPV in relation to unpaid care or vice versa. The full texts of the included articles were then analyzed using framework analysis, an approach often applied in interdisciplinary health research [20]. We read the full texts, extracting findings and mapping them into an analytical framework based on the Gender and COVID-19 matrix. A gender analysis matrix is a systematic approach to organizing information through the use of a gender framework and topic-specific domains [21]. The Gender and COVID-19 matrix was developed, based on previous gender matrices, as an approach to documenting the structural determinants of pandemic-related inequities [21]. It includes gender domains, reflecting structural determinants of gender inequality (norms, access to resources, decision-making power, etc.), and topic domains listed vertically (originally, access to care, etc.). Intersecting cells include prompting questions such as ‘What are the barriers to accessing care? How do they affect genders differently?’. The Gender and COVID-19 Matrix has been applied to conduct media analysis of the gendered effects of COVID across multiple countries [22] as well as to related topic areas, such as the equity impacts of pandemic travel measures [23]. In this scoping review, we applied the gender domains as the codes for the framework analysis and the topics of the review – unpaid care and IPV – as the cases. Sub-questions were then created with each intersecting cell; for example, ‘How did access to resources affect gender distribution of unpaid care during the COVID-19 lockdown?’ In reviewing the literature, we extracted evidence that answered such questions to complete the matrix domains. In cases where key findings from each case set (i.e. IPV and COVID-19 lockdown or Unpaid Care and COVID-19 lockdown) were assigned to the same domains, the authors analyzed their relationship to each other together. We analyzed all the data in the framework to identify themes common across both cases. These themes were refined and reviewed through discussion among the authors until consensus was reached, and mapped content was organized thematically accordingly.

Policy recommendations coded in the literature were also mapped according to the framework codes. Overlaying policy recommendations in the filled-in gender analysis matrix enabled the authors to identify policies which target similar determinants of unpaid care and IPV during major disease outbreaks and which could be beneficial in addressing the two issues concurrently.

## Results

**Figure 1.**
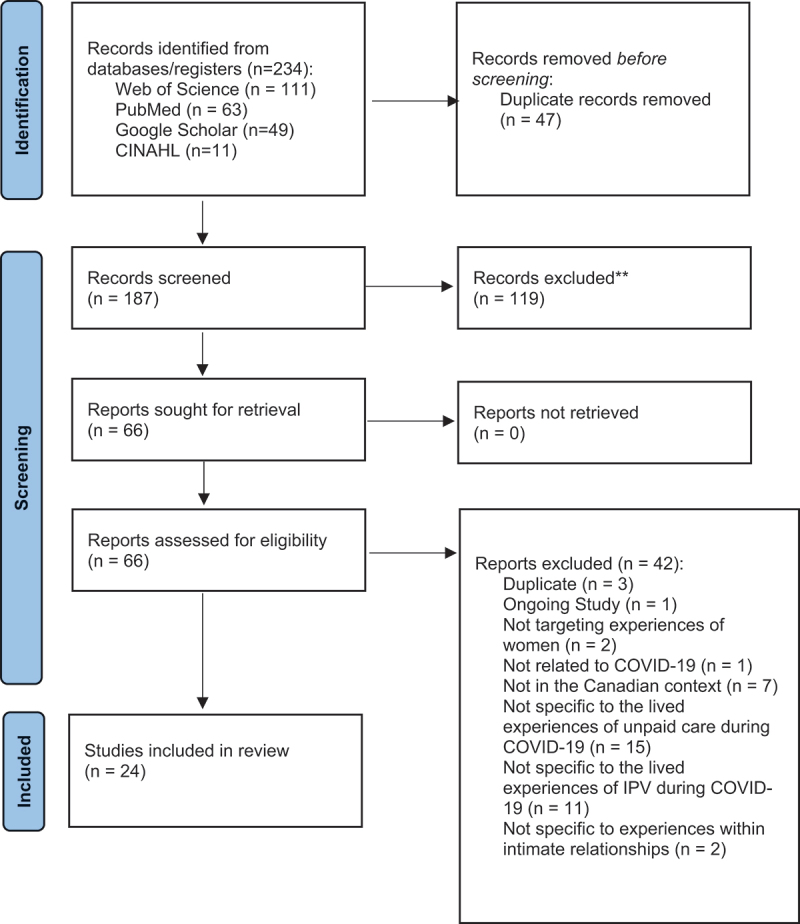
PRISMA schematic showing the total number of studies reviewed as part of the scoping review reported here and reasons for the exclusion of studies. The information included in the schematic is based on that from the PRISMA extension for scoping reviews [18].

**Figure 2.**
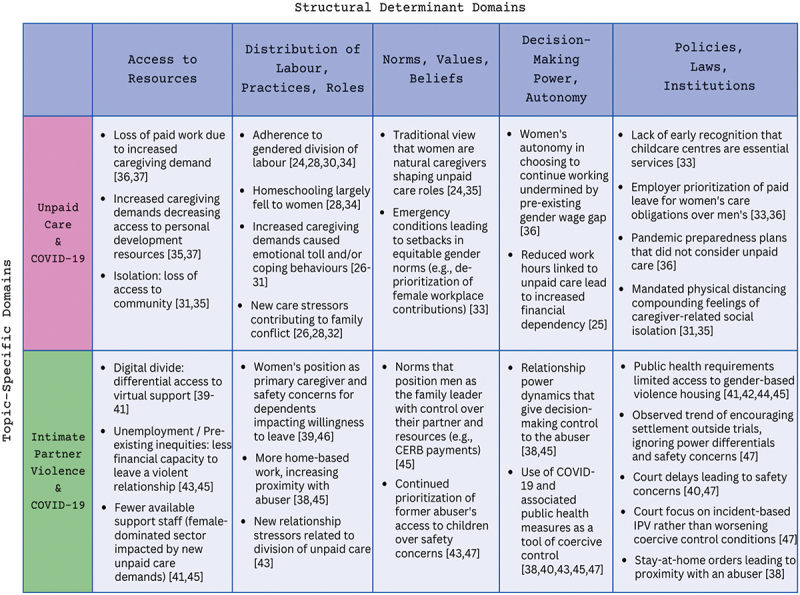
Gender analysis matrix used to explore how structural determinants of gender inequality emerged in experiences of unpaid care and IPV during the COVID-19 lockdown.

**Figure 3.**
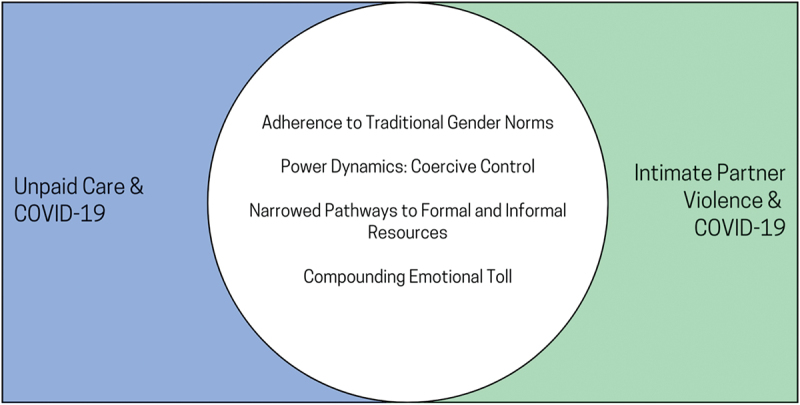
Venn diagram depicting the gendered themes that emerged across both sets of literature.

We identified 14 studies on unpaid care and 10 studies on IPV (24 studies in total – see Appendix B for a full list of included articles) [24–47]. There were an even number of quantitative (10) and qualitative (10) studies and a smaller number (4) of mixed-methods studies. Half (12) presented Canada-wide data, with the remainder focussing on provincial and territory-specific contexts, including Ontario (6), Alberta (2), British Columbia (2), Quebec (1), and the Yukon (1). Research explored the differential experiences of rural residents, immigrant women, and refugee women, with less research on the experiences of trans-women, women in same-sex relationships, and Indigenous women. Appendix B provides an outline of all studies included in this review, the study type (i.e. quantitative, qualitative, or mixed methods), Figure 1 presents the PRISMA schematic of all articles incorporated during the review, Figure 2 presents key findings from the studies mapped onto the gender matrix format, and Figure 3 illustrates the overlapping themes identified through this analysis.

### Adherence to traditional gender norms

Gender analysis of the literature detailing experiences of unpaid care and IPV during the pandemic reveals a traditional patriarchal view of relationship gender roles was frequently present in both experiences. These roles positioned the masculine partner as the household leader, in charge of providing for the family through paid work, while positioning the feminine partner as the primary caregiver, overseeing cooking, cleaning, and new pandemic-related homeschooling arrangements [24,26,27,34]. Changes to the amount of informal care work, fuelling work-family conflict and paid employment instability, led to further entrenchment of traditional gender roles and often brought new stressors to relationships, sometimes fuelling interpersonal conflict [25,28–30]. In some cases, misogynistic views of masculine dominance in relationships resulted in violent assertions of men’s position of power as decision-makers and leaders [45]. The positioning of women as primary caregivers during the pandemic also limited their agency through reduced financial capacity and contributed to unwillingness to leave an abusive situation during the pandemic. This included fears that their children could be separated from them and that dependents would be negatively impacted because of the breaking up of the nuclear family relationship or their reliance on the masculine partner as the ‘breadwinner,’ providing for their shared dependents [31,46].

### Power dynamics featuring coercive control

Traditional gender norms in relationships can lead to power dynamics conferring the greatest decision-making power to the masculine breadwinner. Moreover, for some women who took on a more involved informal care role during the early months of the pandemic, the loss of paid working hours led to increased financial dependency on their partner [43,45]. These gendered relationship dynamics are conducive to the use of coercive control by an abusive partner. In intimate relationships, coercive control describes a pattern of abuse whereby the perpetrator exerts power over their partner to control them and prevent them from exiting the relationship [48]. Qualitative research shows that cases of coercive control worsened during the initial months of the pandemic [31,45]. Examples of coercive control included cases where abusers co-opted their partners’ Canada Emergency Response Benefit (CERB) payments [45]. The reasoning for this action was often founded on the traditional patriarchal understanding that, as the masculine head of the household, they were best equipped to make financial decisions for the family [45]. In Canada, lost work hours as a result of increased caregiving responsibilities due to COVID-19 were coverable under CERB [49]. Given that many women were forced to take a step back from paid work as a result of the imposed demands of caregiving roles, connections can be made between this form of IPV and experiences of unpaid caregiving during the COVID-19 lockdown. Other examples referred to the abusive partner’s use of COVID-19 safety concerns as a reason for controlling movements [43]. These coercive tactics created a dynamic where the abused partner depended on the perpetrator for basic resources and their only source of social contact outside of isolating experiences of caregiving during the pandemic [31]. Additionally, COVID-19-related closures and delays impacted access to justice, particularly family court. These impacts were felt alongside a continued focus by the courts on incident-based physical cases of IPV rather than ongoing patterns of abuse inherent to coercive control dynamics [47]. Such circumstances impacted victims’ ability to obtain separation orders critical to their safety [47].

### Narrowed pathways to resources

Central to both women’s experiences of unpaid care provision and of IPV during the COVID-19 pandemic are limitations placed on access to resources and various formal and informal support services. The early days of the pandemic saw the closure of schools, childcare centres, courts, and anti-violence advocacy and other social support offices [50]. Some civil society organizations transitioned their services to virtual platforms, which presented challenges to connection-building and accessibility for those without safe and consistent access to digital devices [41]. These narrowing pathways to resources came when many needed support most. In many cases, the closure of schools and childcare centres led women to reduce their paid working hours or step away from their own education to meet the care needs of their dependents at home [35,36]. One study, examining the experiences of Yazidi women who recently arrived in Canada as refugees, showed that they were given insufficient computer supplies to allow for both their children’s online schooling and their own English classes, severely limiting their ability to engage with others and find paid employment [35]. These experiences were often associated with rising stress and conflict levels related to home life and financial instability [31]. When read together, the literature indicates that some women faced new economic strains and relationship conflict as a result of their societally imposed caregiving roles during the pandemic. This is significant as past research has connected financial and resource scarcity with a greater risk for relationship abuse [51]. At the same time, in cases where feminine IPV victims felt they needed to exit their relationship, they faced new barriers to balancing employment with childcare and accessing counselling, housing, and anti-violence advocacy resources [45].

Limitations in access to informal supports, such as communities of friends, families, and coworkers, also led to isolation which featured strongly as a theme in the review of both research areas. Isolation was an unintended consequence of the stay-at-home orders designed to stop viral spread at the beginning of the pandemic and was commonly a descriptor attached to the emotional toll of taking on additional burdens of care responsibilities alone and the experience of IPV [45]. One study respondent caring for children during the COVID-19 lockdown while isolated at home with an abusive partner expressed that ‘Having somebody to talk to is so important. I feel like especially when you stay home, with the kids, it’s so lonely.’ [31] p.40]. These accounts of isolation during the early months of the COVID-19 pandemic indicate interactions among different emotional strains, worsening experiences of stressors, and emerging feelings of helplessness. In some cases, imposed isolation brought by COVID-19-related public health measures was weaponized to separate those experiencing violence from friends, family, and social support to take them further under the abuser’s control [38,43]. In this way, isolation can also be an additional barrier to exiting an abusive relationship. This was especially true for those experiencing IPV living in remote and rural locations in Canada through the pandemic [45]. Perceived and imposed isolation contributed to feelings of hopelessness and resignation to the abuse [45].

### Compounding emotional toll

While not a structural determinant, both sets of literature described the effects of these determinants, particularly on women’s wellbeing. Stressors related to unpaid care and IPV for women during the pandemic often compounded to worsen mental health states. Feelings of isolation and moral distress associated with balancing childcare responsibilities with essential work and feeling left behind by the public health response to the pandemic were regularly cited as emotional responses to the circumstances of the pandemic [28,33,36,38]. While some women were able to safely cope with emotional tolls stemming from the gendered impacts of the pandemic by engaging in activities like connecting with the outdoors through exercise or community gardening groups, others turned to substance use [35,38,45]. Substance use is a social activity, and one study observed that an increase in the feminine partner’s substance use while coping with childcare responsibilities during the COVID-19 lockdown regularly led to her relationship partner joining in [28]. Substance use in relationships is a known risk factor for IPV [52], so this response to new emotional tolls associated with increased caregiving responsibilities may be an indirect gendered impact lending to IPV stressors.

### Policy recommendations from the literature

The literature provided a number of policy recommendations to address gender inequities underlying experiences of unpaid care and IPV. A number of these focused on challenges related to childcare. Gordon and Presseau argue that efforts should be made to keep childcare centres and schools operating if at all possible, and where the health crisis presents barriers to the continued operation of childcare centres, deployment of rapid disease testing could ensure childcare remains an option for those testing negative [37]. The closure of childcare centres is seen to disproportionately impact the care responsibilities of women [33], so these efforts would allow more equitable care provision and reduce tensions associated with labour division in relationships. Increased financial support for childcare, both pre- and during the pandemic, enabling both parents to work and maintain independent access to financial resources, was suggested as a way to facilitate equitable relationship dynamics so the burden of care does not disproportionately fall to one partner [32,37]. Financial independence can enhance personal agency in relationships and help ensure financial dependency does not feed into inequitable power dynamics that can result in IPV [53].

At the intersection of unpaid care and IPV, Banerjee et al. note funding could also be provided for collaborative long-term programs aimed at addressing the mental health needs of newcomers to Canada who are also informal caregivers [35]. Such programming could increase awareness of caregiver or IPV support services available in Canada, recognizing that newcomers often lack knowledge of such services and access to services in their first language [46]. Community engagement with women newcomers is essential in the development of these services as institutional gaps in resettlement and pandemic response are shown to quietly rely on the informal caregiving contributions of women [35]. Meanwhile, in reference to abusive relationship dynamics between past intimate partners who share custody of children, Koshan et al. recommend greater explicit acknowledgement that coercive control is a form of abuse in the law [47]. This would allow for the prioritization of the safety needs of those experiencing IPV in shared parenting arrangements over the parenting rights of an abusive partner, recognizing that patterns of coercive control can extend to the shared parenting arrangements [43].

A number of studies note the need for anti-violence organizations to continue providing services throughout crises. Such organizations might be supported to maintain multiple mediums of communication, including walk-in services, for those experiencing IPV during stay-at-home orders [38,40,45]. Maintaining the availability of walk-in services for clients through the pandemic appears to be a crucial aspect of support for those without virtual means, those who are unable to access virtual support as a result of the proximity of their abuser, and digital surveillance of devices by controlling partners [38,39,45]. Finally, a report detailing the experiences of anti-violence organizations through the COVID-19 pandemic suggests that greater collaboration is needed between the anti-violence sector and public health authorities [42,44]. Active communication between these actors could help ensure that pathways to support are not shut to those in need during health crises.

## Discussion

This scoping review explored the interplay between the specific gendered experiences of informal care and IPV during the initial months of the COVID-19 pandemic in Canada, revealing how the two issues reflect common structural determinants of gender inequity. While the literature we analyze is specific to Canada, due to the common experience of pandemic measures around the globe, findings have relevance to other jurisdictions and for pandemic preparedness and response more broadly. First, adherence to traditional gender roles meant that the majority of new and increased caregiving responsibilities fell to women, while abusive relationships reflected sexist views of masculine dominance [35,46]. Second, power imbalances emerged in relationships where unpaid care responsibilities decreased hours that could be dedicated to paid work, limiting economic opportunities and increasing financial dependency on a potentially abusive partner [24,45]. Third, narrowed access to formal and informal support in the area of both care provision and the anti-violence sector meant that there were fewer avenues to escape violent situations [31,35,39–41]. Instead, feelings associated with being cut off from government assistance and isolated from peers exacerbated abuse cycles, trapping victims and leading to maladaptive coping behaviours [28,31,38,43]. These analyses reveal how common determinants of gender inequality structure experiences of IPV and changes in unpaid care during pandemics, with various systems of inequity at different levels of society intersecting.

Gaps in early pandemic response policies contributed to the observed secondary impacts on women’s livelihood, safety, and well-being. Despite Canada’s declared commitment to a feminist and gender-based response, the literature maps how policies aimed at reducing the spread of COVID-19 at the start of the pandemic undervalued the role of gender norms in shaping lived experiences. Closing childcare centres and schools without corresponding appropriate support measures that recognize women’s vital contributions to the care economy limited career and personal development for women and contributed to subsequent financial hardship [25,33]. Literature has shown that these effects decrease personal agency, making those impacted vulnerable to abusive power dynamics [54]. Similarly, worsening conditions for coercive control during the pandemic went unaddressed in policies. Imposed physical distancing and quarantine requirements led to reduced access to formal and informal supports like peer groups [40,41,43,47], which have been shown to be vital to exiting a relationship featuring coercive control dynamics [55]. COVID-related court closures and delays also impacted victims’ ability to obtain separation orders critical to their safety [47]. These conditions allow for coercive control tactics and violence as the abused party is kept within the sphere of abusive power dynamics through activities such as shared parenting arrangements [43].

In addressing these policy gaps through a gender analysis lens, it becomes clear that pandemic contingency plans (plans to be enacted in the case of a pandemic) are insufficient in addressing the structural determinants at the root of experiences of unpaid care and IPV. Rather, pandemic preparedness should be an ongoing process that continually seeks to dismantle systems of inequity which are exacerbated during disease outbreaks and strengthen social support services so that they are more steadfast in times of public health crisis. Policy suggestions in the literature provide a starting point for continuously addressing societal inequities as part of pandemic preparedness efforts as well as during the acute phase of health crises. Based on the evidence reviewed here, we propose four further recommendations:

- Expand access to virtual and remote service options to address digital divides in access to support for both caregiving and IPV. This would address the observed differences in access to support for those residing in remote and rural locations [56]. Virtual counselling is also another pathway to support for those who, before the pandemic, faced barriers to accessing in-person care, such as people with disabilities.
- Promote inclusive workplace cultures that value and encourage the equitable involvement of parents of all genders in unpaid caregiving responsibilities. This recognition of the value of unpaid care work could help to elevate the status of informal caregivers of all genders and transform inequitable gender norms.
- Increased consultation, by governments, with organizations working to address gender inequality to enhance intersectional understanding of the issues of unpaid care and IPV. This process could consider the unique experiences of priority populations, including Indigenous people, racialized people, persons with disabilities, and 2SLGBTQ+ people.
- Government subsidies for childcare and increased funding for the anti-violence sector could simultaneously address gaps in support and recognize the inherent value of caregiving and IPV support activities.

These findings examine the experiences of women in Canada during the pandemic. Though this research may share commonalities with findings from different geographic and disease outbreak contexts, gender norms vary in different cultural environments requiring nuanced analysis. The short time frame for this review, covering March to June 2020, also limits findings to experiences of the most acute phase of the pandemic. Though the findings are of interest for future pandemic preparedness and response, they are nonetheless specific to a precise time and circumstances, limiting their generalizability. This review is further limited in its narrow capacity for intersectional analysis. Considering the limited number of studies included which focused on different intersecting identities, we do not attempt intersectional analysis here. Instead, we examine how literature documents effects on women in general. We recognize this is a limitation of the study to be addressed by future research. Technically, the use of the boolean ‘NOT’ operator may have excluded some articles that would otherwise have been included, but was necessary to screen out the thousands of articles using healthcare terms. Finally, the full-text reviews were carried out by one author, with any uncertainties regarding data extraction raised and evaluated by both authors. Having a single author screen the articles may have increased the risk of bias.

## Conclusion

While the impacts of the COVID-19 pandemic on unpaid care and IPV are well-documented, the relationship between the two areas of study is less well understood. Findings from this work contribute to a more developed understanding of the interconnected nature of gendered dynamics at the root of IPV and inequitable division of unpaid care work following the onset of the COVID-19 pandemic. This scoping review reveals that the gendered relationship between unpaid care and IPV during the COVID-19 lockdown was complex and multi-faceted. Public health measures aimed at reducing the spread of COVID-19 did not adequately consider gender and inadvertently led to secondary impacts reflected in women’s experiences of unpaid care burdens and IPV. Analysis shows that the emotional tolls of both experiences compounded to worsen mental health states. The closure of schools, childcare centres, anti-violence office-based supports, and quarantine requirements at social housing locations created new barriers to support without corresponding assistance and led to setbacks in recent advances in gender equity. Inequitable gender norms saw that the greater share of unpaid care in relationships falls to women, limiting their agency and increasing stress. In some cases, these factors made it difficult for women to assert their needs, exacerbating relationship power dynamics, reaffirming the sexist view that women are subordinate to men and resulting in IPV. The fact that IPV victims are left to the care of the anti-violence sector, most often women-run organizations, is a striking feature of intersecting systems of inequity observed to be at play in unpaid caregiving arrangements and experiences of intimate partner violence through the pandemic. The lack of suitable funding for both this sector and caregiver support reflects social gender norms which view women as natural caregivers and, at the same time, devalue their care contributions. Without appropriate resources, the anti-violence sector is saddled with the expectation that women will continue filling these needs in the absence of support. Institutional intervention through policy over time, paired with social change movements, has the power to enact positive change.

## Appendix A. Search strategy

### Phase 1: care economy and COVID-19 scoping review

- Database(s): Web of Science, PubMed, CINAHL Complete
- Search entry:

(‘quarantine’ OR ‘covid’ OR ‘covid-19’ OR ‘coronavirus’ OR ‘2019-ncov’ OR ‘covid pandemic’ OR ‘sars-cov-2’ OR ‘cov-19’ OR ‘2019 pandemic’ OR ‘lockdown’ OR ‘lockdown phase’ OR ‘stay at home order’ OR ‘social distancing’) AND (‘care economy’ OR ‘unpaid work’ OR ‘domestic work’ OR ‘disability’ OR ‘dependents’ OR ‘caregiver’ OR ‘burden of care’ OR ‘childcare’ OR ‘elder care’ OR ‘daycare’ OR ‘household labour’ OR ‘labour divisions’ OR ‘unpaid household labour’) AND (‘women’ OR ‘girls’ OR ‘females’ OR ‘mothers’ OR ‘guardian’ OR ‘trans women’) AND (‘Canada’ OR ‘Canadian’ OR ‘Canadians’ OR ‘in Canada’ OR ‘Canadian provinces’) NOT (‘healthcare’ OR ‘health services’ OR ‘hospital’ OR ‘health facilities’ OR ‘clinical’)

Figure 1. Search strategy used in phase one of the scoping review search on the topic of unpaid care during the early months of COVID-19 in Canada.

### Phase 2: IPV and COVID-19 scoping review

- Database(s): Web of Science, PubMed, CINAHL Complete
- Search entry:

(‘quarantine’ OR ‘covid’ OR ‘covid-19’ OR ‘coronavirus’ OR ‘2019-ncov’ OR ‘sars-cov-2’ OR ‘cov-19’ OR ‘2019 pandemic’ OR ‘lockdown’ OR ‘lockdown phase’ OR ‘stay at home order’ OR ‘social distancing’) AND (‘IPV’ OR ‘domestic violence’ OR ‘partner abuse’ OR ‘intimate partner aggression’ OR ‘gender-based violence’ OR ‘gbv’) AND (‘women’ OR ‘girls’ OR ‘females’ OR ‘mothers’ OR ‘guardian’ OR ‘trans women’ OR ‘migrant women’ OR ‘women of colour’) AND (‘Canada’ OR ‘Canadian’ OR ‘Canadians’ OR ‘in Canada’ OR ‘Canadian provinces’)

Figure 2. Search strategy used in phase two of the scoping review search on the topic of IPV during the early months of COVID-19 in Canada.

Inclusion:

- Research situated in Canada
- Peer-reviewed article
- Published in English
- Examining the timeframe from March to June 2020 during the COVID-19 pandemic unless the article was found to focus on a different time period featuring stringent stay-at-home measures
- Relays experiences of either unpaid care or intimate partner violence
- Specific focus on women’s experiences

Exclusion:

- Research situated outside of Canada
- Not a peer-reviewed article
- Not published in English
- Research examining experiences outside of the COVID-19 pandemic’s March–June 2020 timeframe when social distancing measures and stay-at-home orders were most stringent, with the exception of localized cases of stringent stay-at-home orders
- Does not detail experiences of either intimate partner violence or unpaid caregiving
- Does not focus on women’s experiences
- Examined forms of violence other than that occurring between current or former intimate partners

## Appendix B.

### Basic characteristics of articles used in review

**Table:**

Author(s), Year	Geographic Location	Type of Study	Covers both unpaid care and IPV explicitly or implicitly	Structural Determinants of Gender Inequality Covered
**Review of Unpaid Care During COVID-19 Lockdowns**				
Haney & Barber, [24]	Canada	Quantitative	No	Norms, Values, & Beliefs; Distribution of Labour, Practices, & Roles
Carroll et al., [25]	Ontario	Mixed Methods	Yes	Decision-Making Power & Autonomy
Elgendi et al., [26]	Canada	Quantitative	Yes	Distribution of Labour, Practices, & Roles
Johnston et al., [27]	Canada	Quantitative	No	Distribution of Labour, Practices, & Roles
DesRoches et al., [28]	Canada	Quantitative	Yes	Distribution of Labour, Practices, & Roles
Mathieu & Tremblay, [29]	Quebec	Quantitative	No	Distribution of Labour, Practices, & Roles
Wister et al., [30]	Canada	Quantitative	Yes	Distribution of Labour, Practices, & Roles
Ray et al., [31]	Yukon	Qualitative	Yes	Access to Resources; Distribution of Labour, Practices & Roles; Policies, Laws & Institutions
McDonald et al., [32]	Canada	Quantitative	Yes	Distribution of Labour, Practices, & Roles
Smith et al., [33]	British Columbia	Qualitative	No	Norms, Values, & Beliefs; Policies, Laws, & Institutions
Shafer et al., [34]	Canada	Quantitative	No	Distribution of Labour, Practices, & Roles
Banerjee et al., [35]	Alberta	Qualitative	No (family stress, not IPV)	Access to Resources; Norms, Values, & Beliefs; Policies, Laws, & Institutions
Smith, [36]	British Columbia	Qualitative	Yes	Access to Resources; Decision-Making Power & Autonomy; Policies, Laws, & Institutions
Gordon & Presseau, [37]	Canada	Quantitative	Yes	Access to Resources
**Review of IPV During COVID-19 Lockdowns**				
Mantler et al., [38]	Ontario	Mixed Methods	No	Decision-Making Power & Autonomy; Policies, Laws, & Institutions
Ghidei et al., [39]	Alberta	Qualitative	No	Access to Resources; Distribution of Labours, Practices, & Roles
MacGregor et al., [40]	Ontario	Mixed Methods	No	Access to Resources; Decision-Making Power & Autonomy; Policies, Laws, & Institutions
Michaelsen et al., [41]	Canada	Qualitative	Yes	Access to Resources; Policies, Laws, & Institutions
Mantler et al., [42]	Ontario	Qualitative	No	Policies, Laws, & Institutions
Archer-Kuhn et al., [43]	Alberta, Manitoba, Ontario	Qualitative	Yes	Access to Resources; Distribution of Labour, Practices, & Roles; Norms, Values, & Beliefs; Decision-Making Power & Autonomy
Wathen et al., [44]	Ontario	Qualitative	No	Policies, Laws, & Institutions
Shillington et al., [45]	Ontario	Mixed Methods	No	All
Blair et al., [46]	Canada	Quantitative	Yes	Distribution of Labour, Practices, & Roles
Koshan et al., [47]	Canada	Qualitative	No	Norms, Values, & Beliefs; Decision-Making Power & Autonomy; Policies, Laws, & Institutions
